# Direct Inhibition of GSK3β by the Phosphorylated Cytoplasmic Domain of LRP6 in Wnt/β-Catenin Signaling

**DOI:** 10.1371/journal.pone.0004046

**Published:** 2008-12-24

**Authors:** Shunfu Piao, Sun-Hye Lee, Hyunjoon Kim, Soohwan Yum, Jennifer L. Stamos, Yongbin Xu, Su-Jin Lee, Jaewon Lee, Sangtaek Oh, Jin-Kwan Han, Bum-Joon Park, William I. Weis, Nam-Chul Ha

**Affiliations:** 1 College of Pharmacy and Research Institute for Drug Development, Pusan National University, Busan, Korea; 2 Department of Molecular Biology, College of Natural Sciences, Pusan National University, Busan, Korea; 3 Division of Molecular and Life Sciences, POSTECH, Pohang, Korea; 4 Departments of Structural Biology and Molecular & Cellular Physiology, Stanford University School of Medicine, Stanford, California, United States of America; 5 PharmcoGenomics Research Center, Inje University, Busan, Korea; University of Washington, United States of America

## Abstract

Wnt/β-catenin signaling plays a central role in development and is also involved in a diverse array of diseases. Binding of Wnts to the coreceptors Frizzled and LRP6/5 leads to phosphorylation of PPPSPxS motifs in the LRP6/5 intracellular region and the inhibition of GSK3β bound to the scaffold protein Axin. However, it remains unknown how GSK3β is specifically inhibited upon Wnt stimulation. Here, we show that overexpression of the intracellular region of LRP6 containing a Ser/Thr rich cluster and a PPPSPxS motif impairs the activity of GSK3β in cells. Synthetic peptides containing the PPPSPxS motif strongly inhibit GSK3β *in vitro* only when they are phosphorylated. Microinjection of these peptides into *Xenopus* embryos confirms that the phosphorylated PPPSPxS motif potentiates Wnt-induced second body axis formation. In addition, we show that the Ser/Thr rich cluster of LRP6 plays an important role in LRP6 binding to GSK3β. These observations demonstrate that phosphorylated LRP6/5 both recruits and directly inhibits GSK3β using two distinct portions of its cytoplasmic sequence, and suggest a novel mechanism of activation in this signaling pathway.

## Introduction

The Wnt/β-catenin signaling pathway is essential for normal development, and is inappropriately activated in a number of cancers and other diseases [1]. This signaling pathway functions by regulating the phosphorylation and degradation of the transcription co-activator β-catenin [2]. In the absence of Wnt, β-catenin is phosphorylated by GSK3β in a complex that includes Axin, GSK3β, and β-catenin [2]. Phosphorylated β-catenin is targeted for degradation via phosphorylation-dependent ubiquitination [3], [4]. Wnt stimulation shuts off β-catenin degradation by inhibiting GSK3β in the Axin complex [5]. This inhibition is believed to be the key event in the activation of the Wnt/β-catenin signaling pathway [6], [7].

Wnt/β-catenin signal transduction is triggered at the plasma membrane by two distinct receptors, the serpentine receptor Frizzled, and the single-transmembrane receptor LRP6 or LRP5 (LRP6/5) [7]–[9]. The extracellular ligand Wnt is thought to promote the assembly of Frizzled, LRP6/5, the cytoplasmic protein Dishevelled and the Axin complex, resulting in the sequential phosphorylation of two Ser/Thr residues in each of five cytoplasmic PPPSPxS motifs of LRP6/5 [10], [11]. The PPPSPxS motif was proposed to be dually-phosphorylated by membrane-recruited GSK3β [10] in the Axin complex and membrane-localized CK1γ [12], [13]. The dually-phosphorylated PPPSPxS motifs are known to mediate the interaction between LRP6/5 and the Axin complex [14], which somehow leads to the activation of the Wnt/β-catenin pathway.

It was previously reported that the overexpression of the intracellular region of LRP6 (residues 1417–1613 of the human sequence), a region that contains a Ser/Thr rich cluster and five PPPSPxS motifs, constitutively activated Wnt/β-catenin signaling and potentiated Wnt3a-induced Wnt/β-catenin signaling [15]. This group also reported that the purified intracellular region of LRP6/5 attenuated GSK3β activity by 20% *in vitro*, strongly indicating that this region of LRP6/5 contains the inhibitory sequence targeting GSK3β and is thus responsible for activating the Wnt/β-catenin signaling pathway in cell [16]. Very recently, it was reported that an intracellular region of LRP6, which contains the Ser/Thr rich cluster and the five PPPSPxS motifs, inhibits the phosphorylation of β-catenin by GSK3β, and that an intact PPPSPxS motif is required [17]. Davison et al. reported that a truncated receptor containing a transmembrane domain and a shorter fragment of LRP6 (residues 1460–1505; referred to as miniC), including the Ser/Thr rich cluster and the first PPPSPxS motif, can activate the Wnt/β-catenin pathway [12]. In particular, they observed CK1-dependent phosphorylation of the PPPSPxS motif and the recruitment of Axin to the membrane, identifying these steps as crucial for the activation of the pathway [12]. However, the actual mechanism by which the GSK3β is inhibited in the Wnt signaling pathway has been obscure.

In this study, we examine the actions of the intracellular region of LRP6/5 on GSK3β in Wnt/β-catenin signaling. We find that phosphorylated LRP6 binds to and directly inhibits GSK3β, and that GSK3β can bridge LRP6 and Axin. These findings lead to a reinterpretation of earlier studies and allow us to propose a molecular mechanism for the activation of the Wnt/β-catenin signaling pathway.

## Results

### The LRP6 PPPSPxS motif can inhibit GSK3β both toward β-catenin and glycogen synthase

We hypothesized that the intracellular LRP6 region acts as a direct GSK3β inhibitor in Wnt/β-catenin signal propagation. Within the intracellular region of LRP6, the miniC region of LRP6, especially the PPPSPxS motif, might be the GSK3β inhibitory sequence; replacement of the cytoplasmic domain of LRP6 with this region can induce Wnt/β-catenin signaling even in the absence of the LRP6 extracellular region [12], [13].

**Figure 1:**
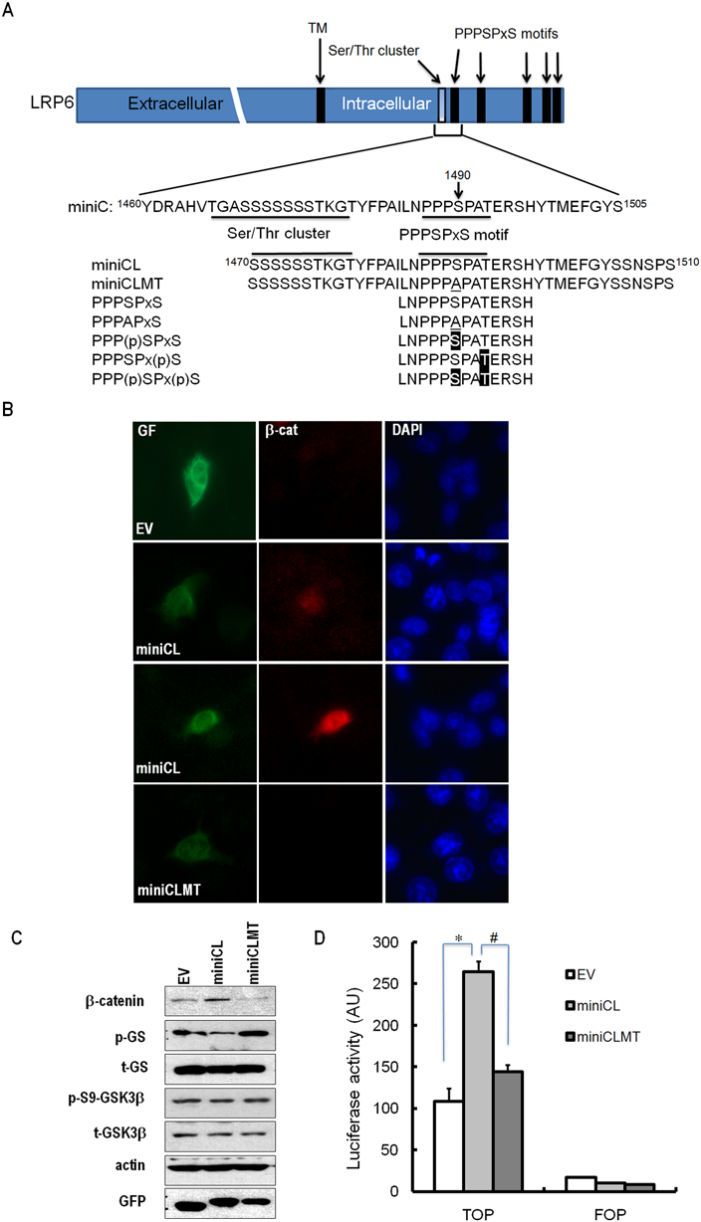
LRP6 constructs and their cytoplasmic overexpression in cell. (A) Schematic representation of the full-length LRP6 and amino acid sequences of LRP6 intracellular fragments used in this study. The Ser/Thr rich cluster and PPPSPxS motif are underlined in each sequence. Phosphorylated residues are highlighted. (B) Cytosolic overexpression of the LRP6 constructs. A549 cells in which control (empty vector, EV, encoding only GFP), miniCL and miniCLMT were transfected as GFP fusion proteins. Control, miniCL and miniCLMT were detected through the green fluorescence from GFP. β-Catenin was detected using an anti-β-catenin antibody, and the nuclei were detected by DAPI staining. (C) Western blotting analysis from the transfected A549 cells used in (B). Labels at the left side of blots indicate the antibody used for detection of the corresponding protein. Endogenous β-catenin was detected by an anti-β-catenin antibody. p-GS indicates the level of the phosphorylated GS by GSK3β, detected by anti-glycogen synthase (Ser641) antibody. p-S9-GSK3β indicates the phosphorylated Ser9 of GSK3β, and was detected by anti-phospho GSK3β (Ser9) antibody. Levels of transfected proteins were detected using monoclonal GFP antibody, indicated by GFP. Total cellular levels of GS and GSK3β were measured by anti-GS antibody and anti-GSK3β antibody, which are indicated by t-GS and t-GSK3β, respectively. Actin is shown as a loading control. (D) Activation of Wnt/β-catenin signaling, which was measured by the Top-flash luciferase activity. Either the top-flash (*TOP*) or fop-flash (*FOP*) luciferase reporter and each construct were transfected into 293 cells. The luciferase activities were measured after 24 h of the transfection. Error bars represent standard deviation from 4 independent experiments. Statistical analysis revealed that overexpression of the miniCL construct significantly activated Wnt/β-catenin signaling compared to that of the empty vector and the miniCLMT construct (* *p*<0.01, # *p*<0.01).

To test our hypothesis, we constructed a GFP-fusion protein based on LRP6-miniCL (Fig. 1A), which is similar to the miniC construct (this protein is called GFP-miniCL; Fig. 1). We also constructed a mutant of LRP6-miniCL containing an Ala substitution at Ser1490 as a GFP-fusion construct (GFP-miniCLMT; Fig. 1). We transfected the constructs into A549 (Figs. 1B and C) and HepG2 cells (Figs. S1 and S2) and analyzed the distribution, expression, and phosphorylation state of each construct, and their effects on β-catenin. Both wild type and mutant miniCL were evenly distributed throughout the cytoplasm (GF; Fig. 1B and Fig. S1). However, only wild-type miniCL dramatically increased the total amount of cellular β-catenin and promoted the translocation of β-catenin into the nucleus (β-cat; Fig. 1B and Fig. S1). We consistently observed an increased level of β-catenin with the miniCL transfection in western blot analyses (β-catenin; Fig. 1C and Fig. S2). These observations are consistent with the results of Cselenyi et al., who used the LRP6 cytoplasmic region containing the Ser/Thr rich cluster and five PPPSPxS motifs, and variant in which the first Ser residue of each PPPSPxS motif is replaced by Ala [17]. To confirm the activation of the Wnt/β-catenin signaling pathway, we measured the transcriptional activity of β-catenin using luciferase in 293 cells. Forced cytoplasmic expression of wild type miniCL, but not the mutant miniCLMT, significantly induced TOP-flash activity (Fig. 1D). These results suggest that the LRP6 miniCL works as an inhibitor of GSK3β regardless of its cellular localization, and that the PPPSPxS motif plays a crucial role in this effect.

The wild-type miniCL, but not the mutant miniCLMT, also suppressed phosphorylation of glycogen synthase (GS; p-GS in Fig. 1C) without the induction of phosphorylation of Ser9 in GSK3β (p-S9-GSK3β; Fig. 1C). Suppression of GS phosphorylation normally occurs when GSK3β is phosphorylated at Ser9 by AKT as a part of the insulin/IGF-1 signaling pathway [18]. Our data indicate that the miniCL protein can inhibit GSK3β independently of AKT activity when this LRP6 fragment is overexpressed in the cytoplasm, implying that it can act as a general inhibitor of GSK3β. Note that under physiological conditions, such inhibition would not occur due to the localization of the LRP6/GSK3β/Axin complex at the plasma membrane (see below).

### Phosphorylated PPPSPxS of LRP6/5 motif directly inhibits GSK3β *in vitro*

**Figure 2:**
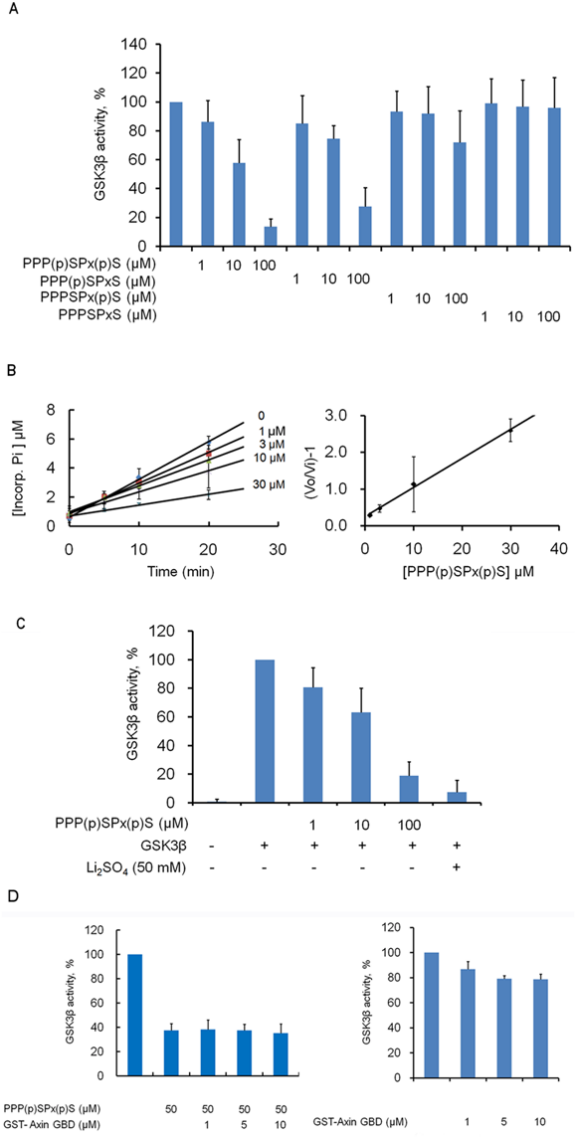
Direct inhibition of GSK3β on the β-catenin N-terminal fragment by LRP6 PPPSPxS peptides. (A) GSK3β activity is inhibited by the LRP6 PPPSPxS peptides depending on the phosphorylation state of the LRP6 motif. The graph is plotted as a percentage of uninhibited GSK3β activity (*Lane 1*) from 4–7 independent experiments; the standard deviation is indicated by error bars. Gels used in this experiment are shown in Fig. S5. (B) Measurement of the inhibition constant (K_i_) of the dually-phosphorylated LRP6 peptide. Varying concentrations (0, 1, 3, 10, and 30 µM) of the dually-phosphorylated LRP6 peptide were used to measure the K_i_ value. The data points are the mean of three independent experiments. The progressive increase in slope indicates that the K_i_ for the peptide is ∼13 µM. (C) The dually-phosphorylated PPPSPxS peptide inhibits the GSK3β-mediated phosphorylation of mouse Axin fragment (residues 512–650). The intensities of the Coomassie-blue stained phosphorylated bands were quantitatively analyzed. The purified Axin fragment (5 µg) was used as a substrate in the same reaction buffer as in (A). The graph is plotted as a percentage of uninhibited GSK3β activity from 3 independent experiments; the standard deviation is indicated by error bars. Gels used in this experiment are shown in Fig. S6. (D) GSK3β is inhibited by the phosphorylated LRP6 motif independently of the Axin GBD protein (*Left*). Binding of Axin GBD to GSK3β is fully saturated at above 2.5 µM of the Axin protein [35], where ∼20% of the GSK3β activity was inhibited by the Axin GBD protein (*Right*). GST-fused mouse Axin GBD (*GST-Axin GBD*, residues 512–530) was used in these experiments. The graph is plotted as relative GSK3β activities from three independent experiments. Error bars indicate the standard deviation. Because the binding constant of Axin GBD domain to GSK3β is about 1 µM, most GSK3β is bound to Axin GBD domain at above 2.5 µM of Axin GBD domain *in vitro*. A representative gel is shown in Fig. S7. Binding of Axin GBD to GSK3β was confirmed by the far western blotting experiment (Data not shown).

Since phosphorylation of the PPPSPxS motif in the miniCL region of LRP6/5 is correlated with the inhibition of GSK3β, we asked whether the PPPSPxS motif can directly inhibit GSK3β *in vitro*. We synthesized one dually-phosphorylated (NPPP**pS**PA**pT**ERSH, where pS or pT designates a phosphorylated Ser or Thr residue), two singly-phosphorylated (NPPP**S**PA**pT**ERSH and NPPP**pS**PA**T**ERSH) and one non-phosphorylated (NPPP**S**PA**T**ERSH) peptides derived from the first PPPSPxS motif of LRP6 (Fig. 1A). To measure the GSK3β activity toward β-catenin *in vitro*, we utilized a β-catenin N-terminal fragment (residues 1–133), whose Ser45 was prephosphorylated by CK1, as a substrate (Ha et al., 2004). Each synthetic peptide was added to a reaction mixture containing the purified GSK3β enzyme and the substrate β-catenin N-terminal fragment. The GSK3β activity was determined through measurement of the intensity of the supershifted (retarded) β-catenin bands (Fig. S3), which represent the phosphorylated product species (Fig. S4), on an SDS-polyacrylamide gel (Figs. S3, S4, S5, S6, S7). The dually-phosphorylated peptide showed a strong inhibitory effect on GSK3β in a concentration-dependent manner (1–100 µM), while the non-phosphorylated peptide did not show any inhibitory effect on the GSK3β activity at concentrations up to 100 µM (Fig. 2A). The two singly-phosphorylated peptides showed distinct inhibitory effects. Whereas the peptide phosphorylated at the second Ser/Thr residue [PPPSPx(p)S; Fig. 2A] exhibited only a marginal inhibitory effect at 100 µM, the peptide phosphorylated at the first Ser/Thr [PPP(p)SPxS; Fig. 2A] exhibited a strong inhibitory effect close to that of the dually-phosphorylated peptide (Fig. 2A). Our results demonstrate that the dually-phosphorylated PPPSPxS motif directly inhibits GSK3β, and that phosphorylation at the first Ser residue plays a more important role. This corresponds well with the results of Zeng et al., who reported that the first Ser residue in the PPPSPxS motif has a more important role in Wnt/β-catenin signaling than the second Ser residue [13]. Since the non-phosphorylated peptide does not inhibit, the inhibition is not due to competition between this potential alternative substrate and the β-catenin substrate.

Based on steady-state kinetic analyses [19], the dually phosphorylated LRP6 peptide competitively inhibits GSK3β with an apparent inhibition constant (K_i_) of 13 µM. Thus, the phosphorylated LRP6 motif is a much stronger inhibitor of GSK3β than the phosphorylated N-terminal fragment of GSK3β (K_i_ ∼700 µM), which is a known pseudosubstrate inhibitor of GSK3β [20] (Fig. 2B).

It was noted above that cytoplasmically expressed miniCL inhibits phosphorylation of GS. To further test whether the dually-phosphorylated PPPSPxS peptide can act as a general inhibitor of GSK3β, we tested its effect on GSK3β-mediated phosphorylation of an Axin fragment (mouse Axin amino acids 512–650), in which Ser614 is the only site phosphorylated by GSK3β *in vitro* and *in vivo* [21]. As shown in Fig. 2C, the phosphorylated PPPSPxS peptides inhibited GSK3β-mediated phosphorylation of the Axin fragment as efficiently as on the primed β-catenin N-terminal fragment substrate. This result is consistent with the observation that the intracellular region of LRP6 decreased GSK3β phosphorylation of an unprimed substrate Tau protein and the primed substrate full-length β-catenin *in vitro* [16]. In addition, the phosphorylated PPPSPxS peptides did not show any inhibitory effect on CK1 when we use the unphosphorylated β-catenin fragment as a substrate (data not shown), confirming that the phosphorylated PPPSPxS peptide inhibits only GSK3β. Collectively, these data suggest that the phosphorylated PPPSPxS motif of LRP6/5 can act as a general inhibitor of GSK3β *in vitro*.

### Axin-bound GSK3β is inhibited by the phosphorylated LRP6 PPPSPxS motifs

We next sought to determine if the phosphorylated LRP6 motifs are capable of inhibiting GSK3β in the Axin complex. An *in vitro* kinase assay with Axin-bound GSK3β was performed by adding the purified GSK3β binding domain (GBD) of Axin to the reaction mixture. Although full-length Axin increases GSK3β activity toward full-length β-catenin through its scaffolding of GSK3β and β-catenin [22], the Axin GBD domain itself slightly inhibited GSK3β (Fig. 2D), which is consistent with the reduced activity of Fratide-bound GSK3β [23]. As shown in Fig. 2D, the dually-phosphorylated PPPSPxS peptide inhibits Axin-bound GSK3β similarly to free GSK3β. This result is consistent with the crystal structure of GSK3β complexed with Axin, which shows that Axin does not occupy the active site of GSK3β [22]. The *in vitro* inhibition of Axin-bound GSK-3β indicates that the phosphorylated PPPSPxS motifs of LRP6/5 can be an *in vivo* inhibitor of GSK3β in Wnt/β-catenin signaling.

### Microinjection of the peptide containing the phosphorylated PPPSPxS motif into the *Xenopus* embryo potentiates Wnt/β-catenin signaling

**Figure 3:**
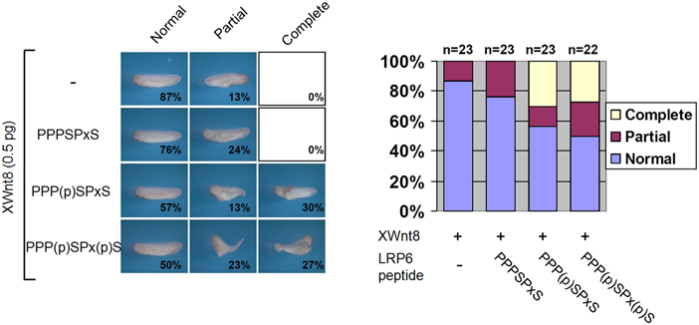
The effect of the injected PPPSPxS peptides to the *Xenopus* embryos. *Xenopus* Wnt8 protein (*XWnt8*; 0.5 pg) was co-microinjected with each peptide (1 ng) into the ventrovegetal region of *Xenopus* embryos. Representative embryo pictures are shown in the left panel, and the diagram shows the ratio of the phenotypes (normal shape, partial and complete second axis duplication) in the right panel. Although the injected peptides would be diluted and degraded as the cells divide due to lack of the *de novo* biosynthesis, the dually and singly-phosphorylated PPPSPxS peptides dramatically increased partial and complete second body axis.

It is well established that Wnt signaling reduces GSK3β activity during development of the *Xenopus* embryo [24], and that the suppressed activity of GSK3β induces a second body axis in the *Xenopus* embryo in the response to Wnt [25]. To examine the *in vivo* effect of the PPPSPxS motif on Wnt/β-catenin signaling, we injected the synthetic peptides into *Xenopus* embryos, along with a small amount of *Xenopus* Wnt8 that is insufficient to induce second body axis formation on its own. The dually-phosphorylated peptide [PPP(p)SPx(p)S; Fig. 3] strongly induced the second body axis, whereas the non-phosphorylated peptide [PPPSPxS; Fig. 3] did not. The peptide phosphorylated at only the first Ser residue [PPP(p)SPxS; Fig. 3] induced the second body axis with an efficiency comparable to that of the dually-phosphorylated peptide. This result indicates that the phosphorylated PPPSPxS motif can induce Wnt signaling *in vivo*. Although the amount injected is likely to be higher than physiological concentrations, it is interesting that microinjection of each peptide in the absence of *Xenopus* Wnt8 did not induce a second body axis (data not shown). It is likely that a slight reduction in the GSK3β activity due to the activity of the exogenous Wnt on the endogenous LRP6/5 is required for the peptide to cause a sufficient inhibition of GSK3β to trigger Wnt/β-catenin signaling. Moreover, even if the amount of peptide injected is well above physiological concentrations, the effect is specific to the phosphorylated peptide. Thus, the peptide injection experiments, when considered with the experiments using purified proteins, indicate that the phosphorylated PPPSPxS motif directly inhibits GSK3β and leads to the activation of β-catenin.

It was recently reported that injection of a recombinant portion of the LRP6 cytoplasmic region containing the Ser/Thr rich cluster and the five PPPSPxS motifs induced a second body axis as effectively as the phosphorylated PPPSPxS peptide in the absence of the exogenously-added Wnt ligand, even when the LRP6 protein was not prephosphorylated by GSK3β and CK1 [17]. It seems likely that the longer fragment of the LRP6 cytoplasmic region is phosphorylated by endogenous kinases in the embryo, and has a stronger inhibitory effect on GSK3β due to the multiple PPPSPxS motifs. Also, as we show below, the Ser/Thr-rich region upstream of the PPPSPxS motifs, may have an important role in this process.

### GSK3β preferentially binds to the phosphorylated intracellular region of LRP6

The evidence above suggests that the phosphorylated PPPSPxS motifs of the LRP6/5 intracellular region competitively inhibit GSK3β. The intracellular region of LRP6/5 has been implicated in binding both GSK3β and Axin [6], [12], [13], [16]. Whereas GSK3β bound to LRP6 or 5 in the absence of Axin in yeast two-hybrid experiments [13], [16], GSK3β dramatically increased the interaction of Axin with LRP6 or 5 in cells and *in vitro* [6]. Moreover, both the GBD and DIX domains of Axin were present in constructs that interacted with LRP5 in yeast two-hybrid experiments [6]. The Axin-binding site found in vertebrate GSK3 [22] is conserved in the yeast homolog. These observations suggest that GSK3 can associate directly with LRP6 and bridge LRP6 to Axin.

**Figure 4:**
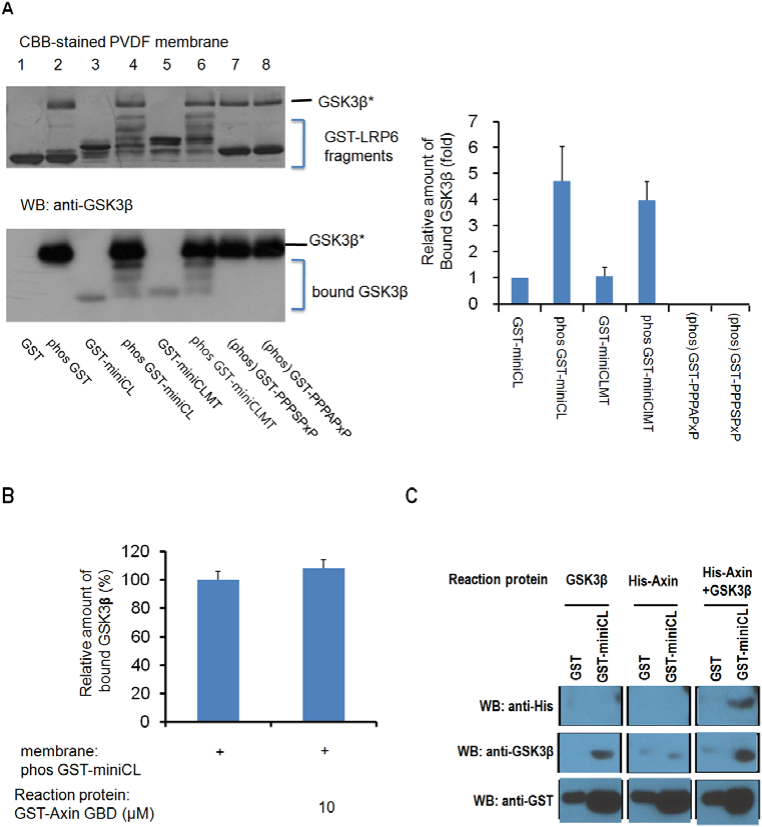
Phosphorylation-dependent binding of the LRP6 intracellular region to GSK3β in the absence and presence of Axin. (A) GST (*Lane* 1), GST-fused miniCL (*Lane* 3; GST-miniCL), GST-fused miniCLMT (*Lane* 5; GST-miniCLMT), and their phosphorylated forms (*Lanes* 2, 4, 6; indicated by “phos”) were loaded onto an SDS-polyacrylamide gel, and then transferred to PVDF membrane (*Left top*). Because GST-fused PPPSPxS and PPPAPxS were not phosphorylated by GSK3β and CK1 (data not shown), we only applied the GSK3β- and CK1-treated GST-PPPSPxS or its mutant form [*Lanes* 7, 8; indicated by (phos)] to the gel, since the untreated forms would be redundant. The phosphorylated protein bands show an upshifted mobility on the gel. The PVDF membrane was incubated with 1 µg/ml of GSK3β to allow GSK3β to bind to the proteins in the membrane. The bound GSK3β was visualized on the transferred proteins by western blotting using an anti-GSK3β antibody (*Left bottom*), and the GSK3β protein used to obtain the phosphorylated forms of each protein is indicated by GSK3β*. The band intensities of bound GSK3β were measured from the three independent experiments (*Right*). (B) The Axin GBD domain does not affect the binding of GSK3β to the miniCL region of LRP6. The phosphorylated GST-fused miniCL was transferred to PVDF membrane, and the membrane was incubated with GSK3β (0.1 µg/ml) and GST-fused Axin GBD domain protein (0, 10 µM). Error bars indicate the standard deviation from three independent experiments. (C) GSK3β mediates the binding of an Axin fragment to LRP6 miniCL region. A His-tagged Axin fragment (*His-Axin*; residues 512–650) including the GBD and the β-catenin binding domain (BBD) was tested if it directly binds to LRP6 miniCL region or its binding is mediated by GSK3β. Fifteen µg of GST-miniCL was transferred to a PVDF membrane, and the membrane was incubated with GSK3β (2 µg/ml) and/or His-Axin (12 µg/ml) in TBST containing 1% skim milk. The Axin fragment was bound to LRP6 miniCL only in the presence of GSK3β.

We first investigated whether the intracellular region of LRP6 binds to GSK3β in a phosphorylation-dependent manner. To detect binding, we used recombinant GST-fused LRP6 fragments (miniCL, miniCLMT, PPPSPxS, PPPAPxS; Fig. 1A) and GSK3β proteins in a far western binding assay. This assay is appropriate as the LRP6 cytoplasmic region is expected to be natively unstructured and therefore should not suffer from denaturation artifacts. Prior to the binding reaction, the GST-fused LRP6 fragments were phosphorylated by GSK3β and CK1 and transferred to a PVDF membrane. The membrane was incubated with GSK3β, and bound GSK3β was detected with an anti-GSK3β antibody. GSK3β bound to GST-miniCL and GST-miniCLMT, but not GST-PPPSPxS or GST-PPPAPxS (Fig. 4). Comparison with LRP6 that had not been phosphorylated revealed that phosphorylation enhanced the binding by four to five fold (Fig. 4A *Right*). Since the S1490A mutation does not affect binding, these results suggest that the Ser/Thr rich cluster present in miniCL plays more important role in mediating the phosphorylation-dependent interaction of LRP6 with GSK3β. Also, given the measured K_i_ value of the single PPPSPxS motif, it was expected that the single PPPSPxS motif might exert a limited role in recruiting the GSK3β.

To investigate whether the Axin-bound GSK3β is able to bind to the phosphorylated LRP6 fragments (miniCL), we incubated GSK3β with excess Axin GBD protein to saturate its binding to GSK3β and then tested the binding of GSK3β and LRP6 using the same far western blot analysis (Fig. 4B). Binding of GSK3β to the LRP6 fragment was not affected by the presence of Axin, indicating that the Axin GBD, GSK3β, and LRP6 miniCL proteins form a ternary complex *in vitro*. Furthermore, we observed that an Axin fragment including the GBD was bound to the LRP6 miniCL fragment only when GSK3β was co-incubated (Fig. 4C), demonstrating that GSK3β mediates the association of the Axin fragment with the LRP6 intracellular region. Taken together, we propose that the GSK3β plays an important role in mediating Axin binding to LRP6/5 *in vivo* mainly through the Ser/Thr rich cluster of LRP6/5.

## Discussion

Here we have provided evidence that the phosphorylated PPPSPxS motif of LRP6/5 directly inhibits GSK3β. The dually-phosphorylated PPPSPxS motif acts as a competitive inhibitor with a K_i_ of 13 µM. Given this K_i_ value, the inhibitory effect of phosphorylated LRP6 on GSK3β is relatively low compared to other cytoplasmic protein inhibitors, such as XIAP (K_i_ toward caspase-3 ∼2 nM [26]), although it is a much stronger inhibitor than the phosphorylated N-terminal segment of GSK3β *in trans*. However, the weak inhibitory effect of the phosphorylated LRP6 on GSK3β could become important when GSK3β and LRP6 are both localized to the membrane, which would bring the enzyme and its inhibitor into close proximity. Moreover, the five PPPSPxS motifs could enhance the avidity of the interaction with GSK3.

Cselenyi et al. (2008) found that the recombinant, nonphosphorylated LRP6 intracellular region at ∼4 µM can inhibit β-catenin phosphorylation by GSK3β, but not Tau, another GSK3β substrate, which led these authors to propose that GSK3β phosphorylates LRP6 and that the phosphorylated species somehow specifically interferes with the β-catenin–GSK3β interaction rather than inhibiting the kinase. They argue that the inhibition of GSK3β observed by Mi et al. (2006) may have been due to the use of supraphysiological concentrations, although the concentrations used in that study were not reported. We have shown that phosphorylated LRP6 inhibits GSK3β activity towards another substrate, Axin, in a purified system (Fig. 2C), arguing against a β-catenin specific inhibitory activity. We also see inhibition of glycogen synthase when the LRP6 cytoplasmic fragment is overexpressed in cells. Although in this case a free cytosolic LRP6 motif is clearly not physiological, taken with the other data it is clear that the phosphorylated motif can act as a competitive inhibitor of GSK3β. The 13 µM K_i_ is consistent with the findings of Cselenyi et al. when the avidity of the 5 PPPSPxS motifs is considered. Although the relevant concentration *in vitro* is not known, particularly given the formation of scaffolding complexes at the plasma membrane, it is clear that the phosphorylated motifs act as potent inhibitors.

The cytoplasmic scaffold protein Dishevelled is an indispensible component downstream of Frizzled in Wnt/β-catenin signaling [27]. A large body of evidence suggests that Dishevelled is responsible for the direct recruitment of Axin to the membrane in Wnt/β-catenin signaling, through the interaction of the Dishevelled and Axin DIX domains [28]–[30]. In this study, we found that the miniCL intracellular region of LRP6/5 has two functional modules, each regulated by their phosphorylation state: the Ser/Thr rich cluster appears to contribute to the binding of GSK3β, and the PPPSPxS motifs regulate GSK3β activity. The interaction of the Ser/Thr rich cluster with GSK3β suggests that the Ser/Thr rich cluster of LRP6/5 and Dishevelled may synergize in the recruitment of the GSK3β–Axin complex to the membrane upon Wnt stimulation. Concurrently, GSK3β co-localized with Axin at the membrane would be inhibited by the phosphorylated PPPSPxS motifs of LRP6/5.

**Figure 5:**
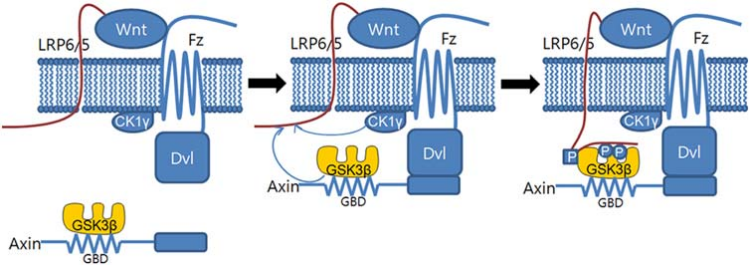
A model for the activation of Wnt/β-catenin signaling. *Left*, simultaneous binding of Wnt to Frizzled (*Fz*) and LRP6/5 leads to recruitment of Dishevelled (*Dvl*) to the Frizzled cytoplasmic region. *Middle*, the Axin complex containing GSK3β is recruited to the membrane via interactions of Axin with Dishevelled, and also the Ser/Thr rich cluster of LRP6/5 with GSK3β in the complex. The membrane-recruited GSK3β, together with the membrane-anchored CK1γ, phosphorylates the Ser/Thr rich cluster of LRP6. Once the Ser/Thr rich cluster of LRP6/5 is phosphorylated, LRP6/5 makes a greater contribution to the recruitment of the Axin complex due to its increased affinity. *Right*, the GSK3β in the Axin complex is inhibited by the phosphorylated PPPSPxS motif in LRP6/5. The phosphorylated Ser/Thr rich cluster is indicated by p in a square, and the phosphorylated PPPSPxS motif is indicated by p in a circle.

Our findings suggest a mechanism to account for the role of phosphorylated LRP6 motifs in the initial events of Wnt/β-catenin signaling (Fig. 5). Binding of Wnt to the Frizzled and LRP6/5 receptors promotes binding of Dishevelled to the cytoplasmic region of Frizzled. This membrane-localized Dishevelled recruits the Axin complex mainly through the Axin-Dishevelled interaction and partly through the LRP6 Ser/Thr rich cluster–GSK3β interaction. The membrane-localized GSK3β in the Axin complex, together with CK1γ, phosphorylates the intracellular region of LRP6/5, including the Ser/Thr rich cluster and the five PPPSPxS motifs. The phosphorylated Ser/Thr rich cluster of LRP6/5 has a higher affinity for GSK3β, and may help to maintain the Axin complex at the membrane in conjunction with the Dishevelled-Axin interaction. Finally, GSK3β in the plasma membrane-localized Axin complexes is inhibited by the phosphorylated PPPSPxS motifs of LRP6/5. According to this model, the cytoplasmic GSK3β molecules involved in insulin/IGF-1 signaling would not be recruited to the membrane because of the lack of the Dishevelled-Axin interaction. As such, this model is compatible with the observation that free cytoplasmic GSK3β is not inhibited in response to Wnt stimulation [18], [31], thereby insulating the insulin/IGF-1 signaling from the Wnt/β-catenin signaling pathway.

It was previously suggested that the roles of phosphorylated PPPSPxS motif are to recruit β-catenin [17] and Axin [6], [12], [13], [16], which would lead to inhibition of β-catenin phosphorylation by GSK3β. Fluorescence resonant energy transfer between tagged proteins suggested that the LRP6 cytoplasmic region binds to β-catenin in the cell leading to the proposal that inhibition of β-catenin phosphorylation results from the association of the LRP6 region with β-catenin, not from the inhibition of GSK3β [17]. However, we find that intracellular region of LRP6 directly binds to GSK3β through the Ser/Thr rich cluster and thereby mediates the recruitment of Axin, suggesting that β-catenin associates indirectly with LRP6 through GSK3β and Axin. Likewise, the ability of GSK3β to bind simultaneously to LRP6 and Axin explains earlier co-immunopreciptiation data [6] that were used to conclude that LRP6 and Axin interact directly. In summary, our results show that the phosphorylated PPPSPxS motif of LRP6 acts as a direct inhibitor of GSK3β in Wnt/β-catenin signaling, and suggest a mechanism for stabilization of β-catenin upon activation of cell surface Wnt receptors.

## Materials and Methods

### Plasmids for transfection

DNA fragments encoding LRP6 miniCL (residues 1470–1510 of human LRP6) were obtained from a human cDNA library by PCR, and inserted into the pEGFP-C1 vector (Clontech) using *HindIII* and *BamHI* sites of to generate GFP-tagged protein, resulting in pEGFP-miniCL. The LRP6 mutant (S1490A) was generated by PCR-mediated mutagenesis, resulting in pEGFP-miniCLMT.

### Antibodies

Antibodies were used according to the manufacturer's instructions. Antibodies used include mouse monoclonal anti-β-catenin (Santa Cruz), anti-GFP (Santa Crutz), and rabbit polyclonal anti-GSK3β (Cell signaling), anti-phospho GSK3β (Ser9) (Cell signaling), anti-phospho LRP6 (Ser1490) (Cell signaling), anti-phospho-glycogen synthase (Ser641) (Cell signaling), anti-GS (Cell signaling), anti-phospho-β-catenin (Ser45) (Cell signaling), and anti-phospho-β-catenin (Ser33/37/Thr41) (Cell signaling).

### Transfection and protein analysis

pEGFP-miniCL and pEGFP-miniCLMT vectors were transfected into A549 cells and HepG2 cells using jetpei according to the manufacturer's protocol (Polyplus transfection). In brief, 2 µg of DNA and jetpei mixtures were added to A549 cells and HepG2 cell and incubated for 3 h under serum-free conditions, and then equal volumes of DMEM containing 20% serum were added. After 24 h incubation, cells were harvested using RIPA (containing protease cocktail). Subsequently, 20 µg of protein lysates were applied to SDS-PAGE. After transferring the proteins to a PVDF membrane, samples were incubated with the appropriate antibodies, according to standard western blot protocol.

### Luciferase assay

To address the transcriptional activity of β-catenin, TOP-flash (responsive luciferase vector) and FOP-flash (unresponsive vector), which were kindly provided by Drs. B. Vogelstein and K. Kinzler (Johns Hopkins Univ.), were transfected into 293 cells. Using 10 µl of cell lysate, we performed the luciferase assay using the luciferase assay kit (Promega).

### Immunofluorescence staining

Transfected cells were fixed with 100% methanol for 20 min at −20°C. After washing with PBS and subsequent blocking with blocking buffer (PBS+0.05% BSA), fixed cells were incubated with anti-β-catenin antibody for 2 h at room temperature. After washing with PBS, cells were incubated with a rhodamine-conjugated anti-mouse antibody for 2 h. To visualize nuclei, cells were stained with DAPI for 5 min. After washing with PBS, expression of β-catenin, GFP, and DAPI were analyzed using fluorescence microscopy.

### Expression and purification of recombinant proteins

The catalytic domains of mouse GSK3β (residues 27–393) and CK1ε (residues 1–319) were expressed and purified as previously described [32]. The N-terminal region (residues 1–133) of β-catenin was expressed in *Escherichia coli* as a GST-fusion protein with a TEV protease cleavage site and then purified using Glutathione-agarose. Subsequently, the GST tag was cleaved using recombinant TEV protease and was removed by repeated HiTrapQ anion-exchange chromatography. The N-terminal region of β-catenin was then phosphorylated by incubation with the catalytic domain of CK1ε (0.28 mg/ml) at 37°C for 20 min in 50 mM Tris buffer (pH 8.0) containing 10 mM MgCl_2_, 10 mM 2-mercaptoethanol, and 2 mM phenylmethylsulfonylfluoride. The phosphorylated β-catenin fragment was then further purified using HiTrapQ anion-exchange chromatography to remove the CK1ε enzyme. The mouse Axin GBD (residues 512–530), and variants of human LRP6 (miniCL, residues 1470–1510; miniCLMT, residues 1470–1510 with substitution at Ser1490 with Ala; LRP6 PPPSPxS, residues 1485–1497; and LRP6 PPPAPxS, residues 1485–1497 with the substitution at Ser1490) were expressed in *E.coli* as GST-fusion proteins and purified using Glutathione-agarose and a HiTrapQ anion-exchange chromatographic column. The final buffer for the GST-fusion proteins was changed to 20 mM Tris buffer (pH 8.0) using Centriprep (10 kDa cutoff; Millipore). His-tagged Axin fragment (512–650 in the mouse Axin numbering) was expressed in *E.coli*, and purified using Ni-NTA affinity and HitrapQ anion exchange chromatographic columns.

### Peptide synthesis

Peptides for inhibition assays were prepared by solid-phase synthesis. The amino acid sequence of each peptide is shown in Fig. 1A. The N-terminus and C-terminus of all peptides were modified by biotinylation and amidation, respectively. Biotinylation was accomplished using sulfo-NHS-SS-biotin (Pierce), which allowed reductive cleavage of the 15-mer from the biotin group. The identity and purity of the synthesized peptides were confirmed by mass spectrometry.

### *In vitro* GSK3β activity assay in the presence of synthetic peptides

To measure the GSK3β activity in the presence of each synthetic peptide dissolved in 10% dimethylsulfoxide, 7.5 µl of the reaction mixture was incubated for 30 min at 37°C. This reaction mixture contained 50 mM Tris (pH 8.0), 10 mM MgCl_2_, 10 mM 2-mercaptoethanol, 1 mM ATP, 2 ng of recombinant GSK3β catalytic domain, 7 µM of the primed β-catenin N-terminal fragment, and the same volume (0.3 µl) of varying concentrations of each synthetic peptide. After the reaction was stopped by the addition of SDS-loading buffer and immediate boiling, the resultant mixture was applied to a 15% SDS-polyacrylamide gel for analysis. As shown in Fig. S3, S4, S6, and S7, the intensities of the supershifted bands (substrate, product a, product b) on the Coomassie-stained gel were integrated using the program IMAGEQUANT (Molecular Dynamics, USA). To calculate the GSK3β activity, the intensities of the two product bands were combined according to the number of phosphate groups incorporated by GSK3β. The lower band (product a) contains one phosphate group (×1), while the upper (product b) band contains two or three phosphate groups (×2.5). To examine the effect of Axin GBD on the inhibitory role of the dually-phosphorylated peptide toward GSK3β activity, each concentration of GST-fused Axin GBD protein was added in 7.5 µl of the reaction mixture with GSK3β.

### Determination of the K_i_ value of the phosphorylated LRP6 peptide toward GSK3β

Thirty µl of the reaction mixture containing 50 mM Tris (pH 8.0), 10 mM MgCl_2_, 10 mM 2-mercaptoethanol, 1 mM ATP, 8 ng of recombinant GSK3β catalytic domain, 6 µM of the primed β-catenin N-terminal fragment, and varying concentrations of dually-phosphorylated peptide were incubated at 37°C. At each time point, 7.5 µl was taken from the reaction mixture for SDS-PAGE. The GSK3β activity was measured using a Coomassie-stained SDS-polyacrylamide gel, as described above. The initial parts of the activity curves, which show no curvature, were obtained and their slopes were calculated as initial velocities. The apparent K_i_ value was determined according to the equation: (V_0_/V_i_)−1 = [peptide]/K_i_, where V_0_ and V_i_ are the initial velocities of the reaction in the absence and in the presence of the dually-phosphorylated peptide [19].

### *Xenopus laevis* embryo manipulations

*Xenopus laevis* was purchased from Xenopus I and Nasco. Eggs were obtained from *Xenopus laevis* primed with 800 U of human chorionic gonadotropin (Sigma). *In vitro* fertilization was performed as described previously [33], and the developmental stages of the embryos were determined according to Nieuwkoop and Faber [34]. Microinjection was carried out in 0.33×Modified Ringer's (MR) containing 4% Ficoll-400 (GE healthcare) using a Nanoliter Injector (WPI). Injected embryos were cultured in 0.33×MR until stage 8 and then transferred to 0.1×MR until they had reached the appropriate stage.

### Phosphorylation of GST-fusion proteins

To phosphorylate GST-fusion proteins, 15 µg of each GST-fusion protein was incubated for 4 h at 37°C with 0.2 µg of recombinant GSK3β and 0.4 µg of recombinant CK1ε in 10 µl of 50 mM Tris (pH 8.0) buffer containing 5 mM ATP, 10 mM MgCl_2_ and 10 mM 2-mercaptoethanol.

### Far western blotting analysis

GST-fusion proteins (GST-miniCL, GST-miniCLMT, GST-PPPSPxS, GST-PPPAPxS) were subjected to SDS-PAGE and transferred to a PVDF membrane. The membrane was then blocked for 1 h with 5% skim milk in TBST (20 mM Tris (pH 7.6), 137 mM NaCl, 0.05% Tween 20), and rinsed with TBST. Then the membrane was incubated for 3 h with the 1 µg of the recombinant GSK3β in 1 ml of 5% skim milk in TBST, and was washed 3 times with TBST. The binding of GSK3β was detected with rabbit anti-GSK3β antibody and secondary HRP-conjugated goat anti-rabbit IgG (Pierce).

## Supporting Information

Figure S1 Cytosolic overexpression of the LRP6 constructs in HepG2 cells. HepG2 cells in which control (empty vector, EV, encoding only GFP), miniCL and miniCLMT were transfected as GFP fusion proteins. Control, miniCL and miniCLMT were detected through the green fluorescence from GFP. β-Catenin was detected using an anti-β-catenin antibody, and the nuclei were detected by DAPI staining.
(0.91 MB TIF)

Figure S2 Western blotting analysis from the transfected HepG2 cells. Labels at the left side of blots indicate the antibody used for detection of the corresponding protein. The endogenous β-catenin level was detected by an anti-β-catenin antibody. p-GS indicates the level of the phosphorylated GS by GSK3β, detected by anti-glycogen synthase (Ser641) antibody. p-S9-GSK3β indicates the phosphorylated Ser9 of GSK3β, and was detected by anti-phospho GSK3β (Ser9) antibody. Levels of transfected proteins were detected using monoclonal GFP antibody, indicated by GFP. Pan-Ras is shown as a loading control.
(0.05 MB TIF)

Figure S3 In vitro GSK3β activity assay based on band-shifts. The numbers at the right indicate the numbers of the incorporated phosphate groups by CK1 or GSK3β. We used unphosphorylated β-catenin 1–133 region (0) as a substrate, and CK1 and GSK3β proteins were sequentially treated in the reaction buffer used in Fig. 2. The bands for GSK3β substrate is indicated by “substrate”, and the product bands are indicated by “product a” and “product b”. The bands were visualized by Coomassie staining.
(0.16 MB TIF)

Figure S4 In vitro GSK3β kinase assay based on the phospho-specific antibodies against β-catenin. Unphosphorylated β-catenin 1–133 region (0) was used as a substrate, and CK1 and/or GSK3β was treated simultaneously in the same reaction buffer used in Fig. 2. To confirm the inhibitory role of the PPPSPxS peptides, each peptide was added to the reaction mixture. SDS-PAGE was applied to analyze the result. One of gels was stained by Coomassie blue (Top), the other two gels were transferred to PVDF membranes. One membrane was visualized using anti-phospho-β-catenin (Ser45) antibody (Middle), and the other membrane was visualized using anti-phospho-β-catenin (Ser33/37/Thr41) antibody (Bottom). The results are well-consistent with Fig. S1 and Fig. S2, which confirms the fidelity of the in vitro kinase assay used in this study.
(0.23 MB TIF)

Figure S5 Gel figures for Fig. 2A. The bands labeled “substrate” are the prephosphorylated β-catenin 1–133 fragment by CK1. The two product bands are indicated.
(0.49 MB TIF)

Figure S6 Gel figures for Fig. 2C. The bands labeled substrate are the unphosphorylated Axin fragment, and the band labeled product is the Axin fragment harboring phosphorylation at Ser614. Prior to the experiment, we found that the GSK3β-mediated phosphorylation of the Axin fragment can be detected through a band upshift of the fragment on an SDS-polyacrylamide gel. The reaction buffer was the same as in Fig. S2, and the incubation time was 1 hour. In a control experiment, the primed β-catenin (1–133) was used as a substrate using the same amount of GSK3β and reaction buffer, but was incubated for 15 min (See the “control” lanes in the first gel).
(0.22 MB TIF)

Figure S7 A representative gel for Fig. 2D.
(0.11 MB TIF)
