# Supplementary figures and images for: Direct Inhibition of GSK3β by the Phosphorylated Cytoplasmic Domain of LRP6 in Wnt/β-Catenin Signaling
Source: PLoS One. 2008 Dec 24;3(12):e4046. doi: 10.1371/journal.pone.0004046 (PMC2603313; doi:10.1371/journal.pone.0004046)

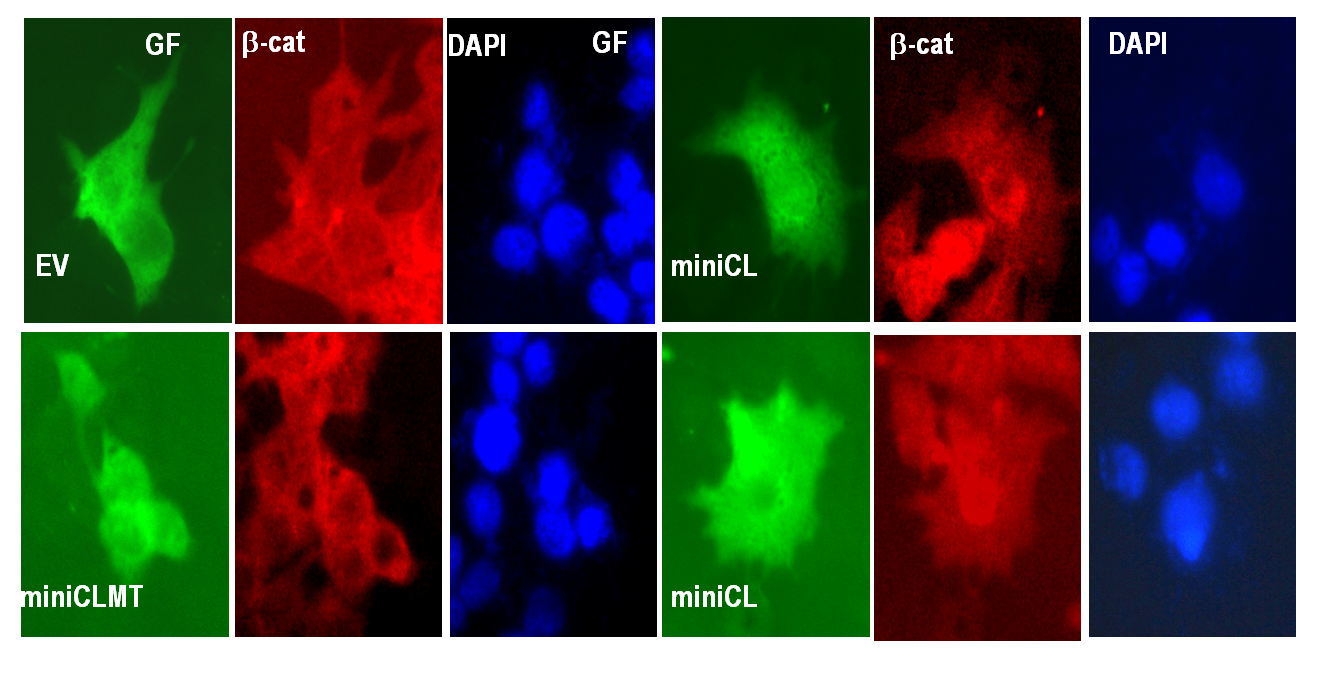

Supplement: Figure S1 — Cytosolic overexpression of the LRP6 constructs in HepG2 cells. HepG2 cells in which control (empty vector, EV, encoding only GFP), miniCL and miniCLMT were transfected as GFP fusion proteins. Control, miniCL and miniCLMT were detected through the green fluorescence from GFP. β-Catenin was detected using an anti-β-catenin antibody, and the nuclei were detected by DAPI staining. (0.91 MB TIF) [file pone.0004046.s001.tif]

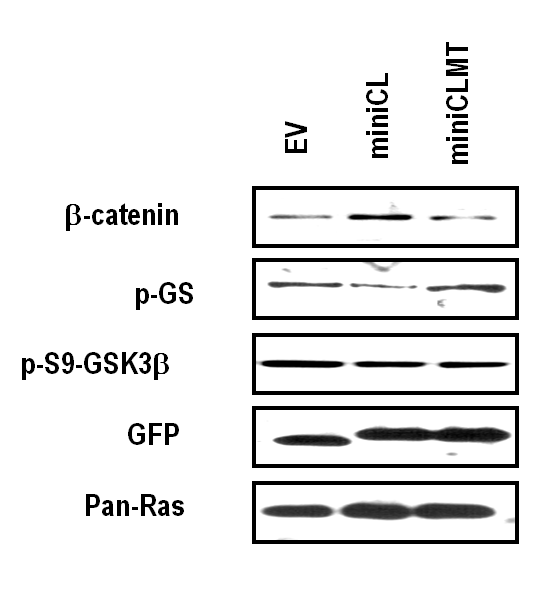

Supplement: Figure S2 — Western blotting analysis from the transfected HepG2 cells. Labels at the left side of blots indicate the antibody used for detection of the corresponding protein. The endogenous β-catenin level was detected by an anti-β-catenin antibody. p-GS indicates the level of the phosphorylated GS by GSK3β, detected by anti-glycogen synthase (Ser641) antibody. p-S9-GSK3β indicates the phosphorylated Ser9 of GSK3β, and was detected by anti-phospho GSK3β (Ser9) antibody. Levels of transfected proteins were detected using monoclonal GFP antibody, indicated by GFP. Pan-Ras is shown as a loading control. (0.05 MB TIF) [file pone.0004046.s002.tif]

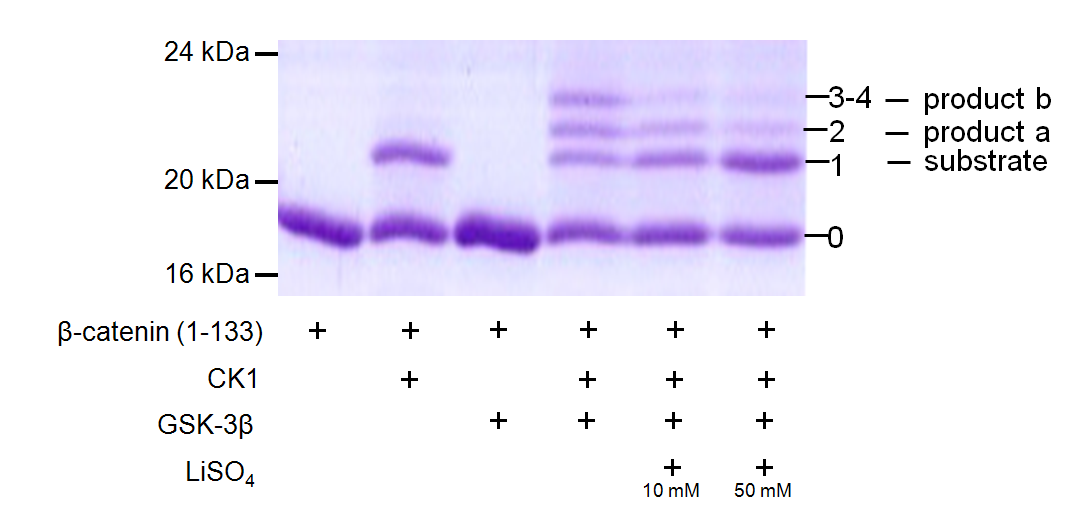

Supplement: Figure S3 — In vitro GSK3β activity assay based on band-shifts. The numbers at the right indicate the numbers of the incorporated phosphate groups by CK1 or GSK3β. We used unphosphorylated β-catenin 1–133 region (0) as a substrate, and CK1 and GSK3β proteins were sequentially treated in the reaction buffer used in Fig. 2. The bands for GSK3β substrate is indicated by “substrate”, and the product bands are indicated by “product a” and “product b”. The bands were visualized by Coomassie staining. (0.16 MB TIF) [file pone.0004046.s003.tif]

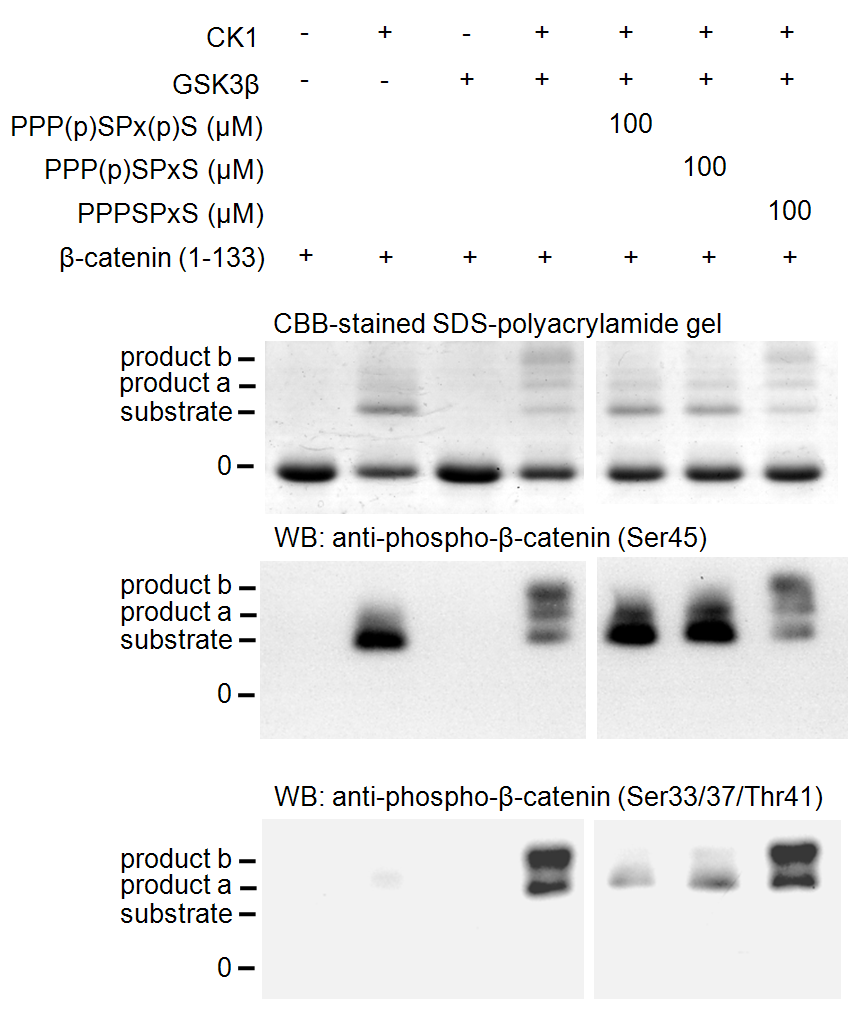

Supplement: Figure S4 — In vitro GSK3β kinase assay based on the phospho-specific antibodies against β-catenin. Unphosphorylated β-catenin 1–133 region (0) was used as a substrate, and CK1 and/or GSK3β was treated simultaneously in the same reaction buffer used in Fig. 2. To confirm the inhibitory role of the PPPSPxS peptides, each peptide was added to the reaction mixture. SDS-PAGE was applied to analyze the result. One of gels was stained by Coomassie blue (Top), the other two gels were transferred to PVDF membranes. One membrane was visualized using anti-phospho-β-catenin (Ser45) antibody (Middle), and the other membrane was visualized using anti-phospho-β-catenin (Ser33/37/Thr41) antibody (Bottom). The results are well-consistent with Fig. S1 and Fig. S2, which confirms the fidelity of the in vitro kinase assay used in this study. (0.23 MB TIF) [file pone.0004046.s004.tif]

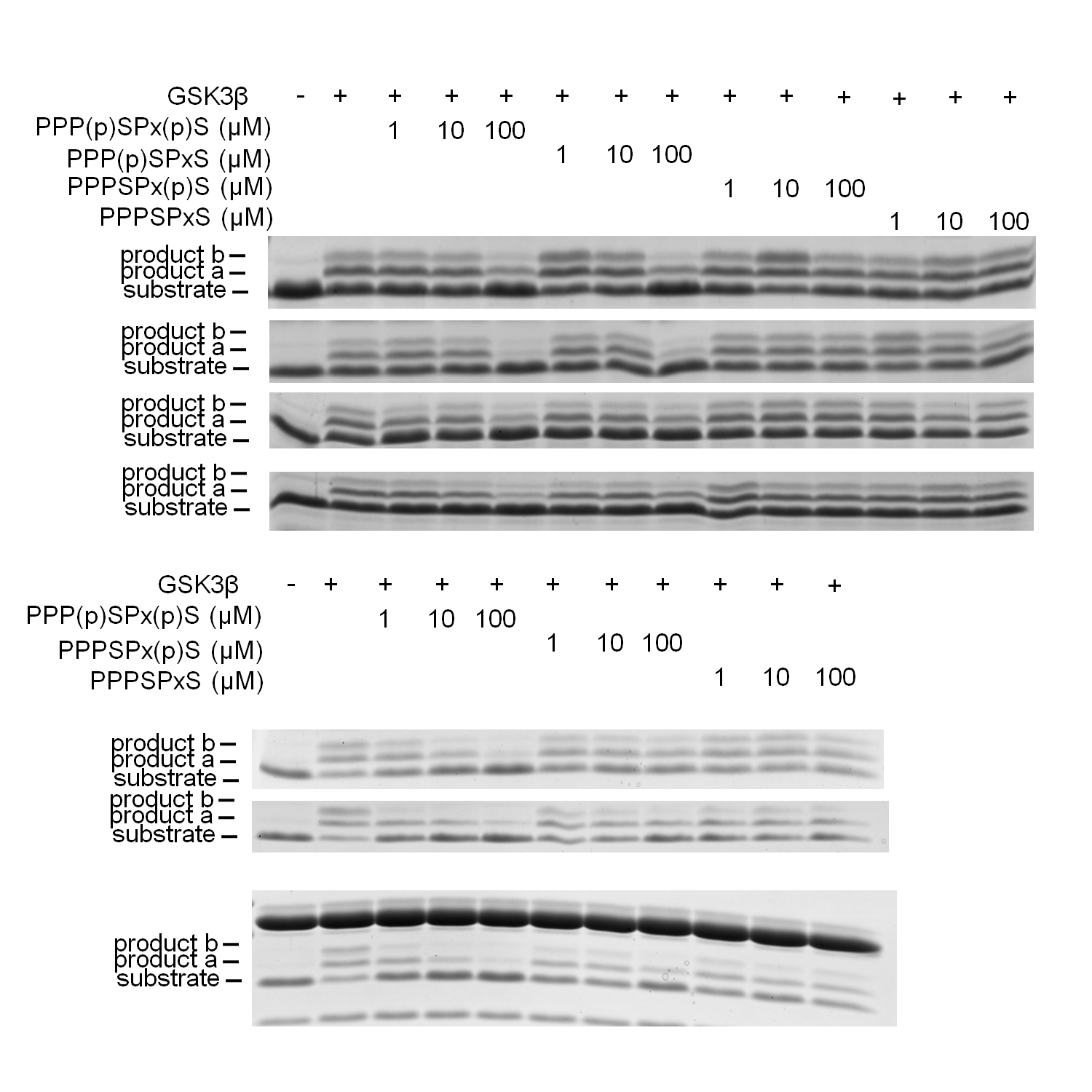

Supplement: Figure S5 — Gel figures for Fig. 2A. The bands labeled “substrate” are the prephosphorylated β-catenin 1–133 fragment by CK1. The two product bands are indicated. (0.49 MB TIF) [file pone.0004046.s005.tif]

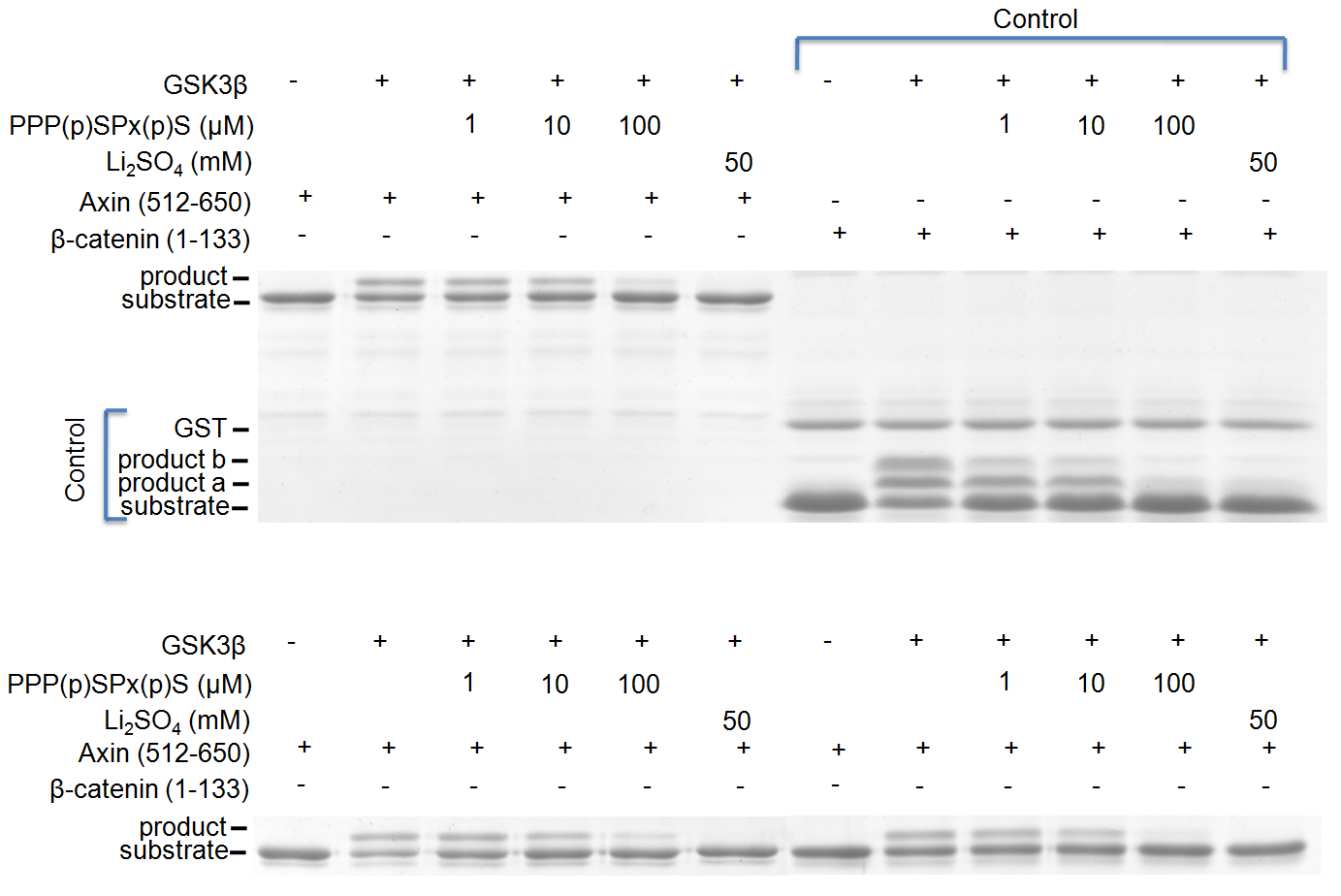

Supplement: Figure S6 — Gel figures for Fig. 2C. The bands labeled substrate are the unphosphorylated Axin fragment, and the band labeled product is the Axin fragment harboring phosphorylation at Ser614. Prior to the experiment, we found that the GSK3β-mediated phosphorylation of the Axin fragment can be detected through a band upshift of the fragment on an SDS-polyacrylamide gel. The reaction buffer was the same as in Fig. S2, and the incubation time was 1 hour. In a control experiment, the primed β-catenin (1–133) was used as a substrate using the same amount of GSK3β and reaction buffer, but was incubated for 15 min (See the “control” lanes in the first gel). (0.22 MB TIF) [file pone.0004046.s006.tif]

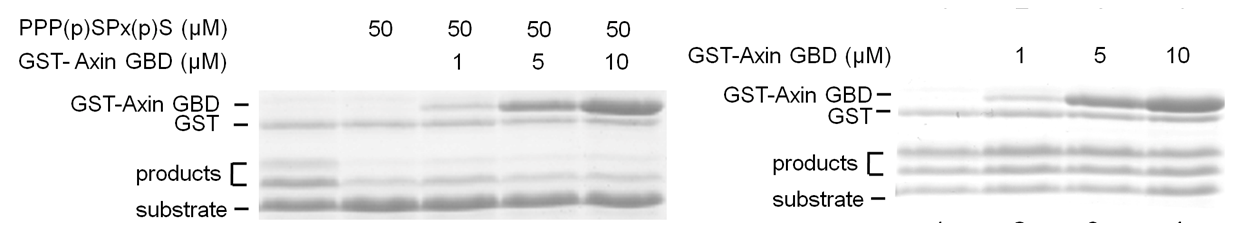

Supplement: Figure S7 — A representative gel for Fig. 2D. (0.11 MB TIF) [file pone.0004046.s007.tif]
